# Supplementary material for: Periodic squeezing in a polariton Josephson junction
Source: Nat Commun. 2017 Nov 6;8:1329. doi: 10.1038/s41467-017-01331-8 (PMC5673906; doi:10.1038/s41467-017-01331-8)
Supplement: Supplementary file 1 — Supplementary Information [file 41467_2017_1331_MOESM1_ESM.pdf]

Supplementary Note 1. Experimental setup and data processing.

The sketch of the experimental setup is shown in Supplementary Fig. 1. The emission of the Ti:Sapphire mode-locked laser is set to $\lambda = 848$ nm, with a pulse duration of $\approx$300 fs, and a repetition rate of 80.5 MHz. The optical pulses subsequently pass through a Fastpulse 5046SC-1/4 electro-optic modulator (EOM) and an IntraAction AOM-802N acousto-optic modulator (AOM). We run in a mode such that the EOM passes only one laser pulse, blocking the rest with an extinction ratio better than 10^3. Subsequently, the AOM completely suppresses all of the attenuated laser pulses outside the time window of $\sim$2.5 µs, as shown on the left of Supplementary Fig. 1. The repetition rate of the single pulses is 64 Hz and is limited by the frame rate of the Hamamatsu ORCA-R2 CCD camera.

After the pulse picking, a single grating pulse shaper is used to reduce the laser spectral width to 0.7 meV. This increases the pulse duration, which nevertheless remains well below than the resolution of the streak-camera (3.4 ps FWHM).

To reduce the time jitter of the streak-camera during the long experimental time, we send a fraction of the exciting laser onto the streak-camera slit and use it as a time reference. All the single photon counts merged into one image clearly showing the presence of Josephson oscillations (Fig. 2a in the main text), confirming the quality of the jitter correction procedure.

For the spatial stabilization of the microcavity sample, the data acquisition is paused every 10 000 pulses, and the real-space luminescence of the sample under nonresonant continuous-wave excitation (shown with green in Supplementary Fig. 1) is recorded with a CCD camera. If the shift of the sample is detected, the position of the cryostat is adjusted using the piezo actuators.

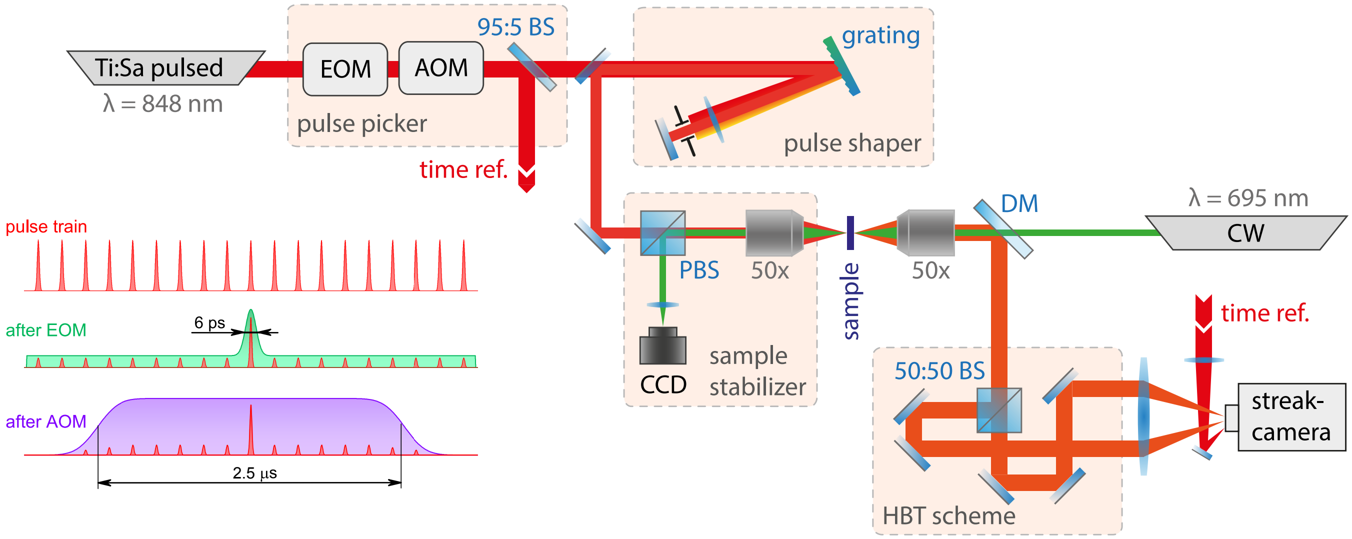

Supplementary Figure 1. **Sketch of the experimental setup.** EOM: electrooptic modulator; AOM: acoustooptic modulator; BS: beamsplitter; PBS: polarizing beamsplitter, DM: dichroic mirror. On the left, the scheme of the pulse picking with a combination of EOM and AOM is shown.

The time-resolved images are processed with a custom photon-counting algorithm. The threshold for photon detection on the CCD was chosen to be high enough in order to resolve the pairs of photons that are close to each

other. Next, for every pulse, the number of photons in the left and the right arm $n_{\mathrm{L,R}}(t)$ are calculated as the number of photons arriving within the time interval $(t - \Delta t/2, t + \Delta t/2)$, where Δt is the duration of the time bin. Increasing Δt increases the total counts per bin however it also decreases the temporal resolution. In the data presented in this paper, $\Delta t = 1.296$ ps, which corresponds to two pixels on the streak-camera screen.

The intensity graph (Fig. 2a in the main text) is calculated as $I_{\mathrm{L,R}}(t) = \langle n_{\mathrm{L}} + n_{\mathrm{R}} \rangle$. The second-order correlation function was calculated as

$$g^{(2)}(t_1, t_2) = \frac{\langle n_1(t_1) \cdot n_2(t_2) \rangle}{\langle n_1(t_1) \rangle \langle n_2(t_2) \rangle}, \tag{1}$$

where $n_{1,2}(t)$ are the photon streams detected by the streak-camera in two arms of the HBT setup and the averaging is performed over the number of laser pulses. The errors of $g^{(2)}(0)$ values are calculated from the standard errors of $\langle n_1 \rangle$, $\langle n_2 \rangle$ and $\langle n_1 \cdot n_2 \rangle$.

Supplementary Note 2. Effect of the occupancy on the light statistics.

In Supplementary Fig. 2 we present the dependence of the second-order correlation function on the total population of the system n_{tot} and the interrelation between the phases of the displacement and squeezing parameters expressed as $\cos(\theta - 2\varphi)$. This data is calculated from Equation (1) of the main text for four different squeezing amplitudes r. It can be seen that for any field amplitude the observed $g^{(2)}(0)$ value can be varied in broad range by varying both the squeezing parameter and the phase relation.

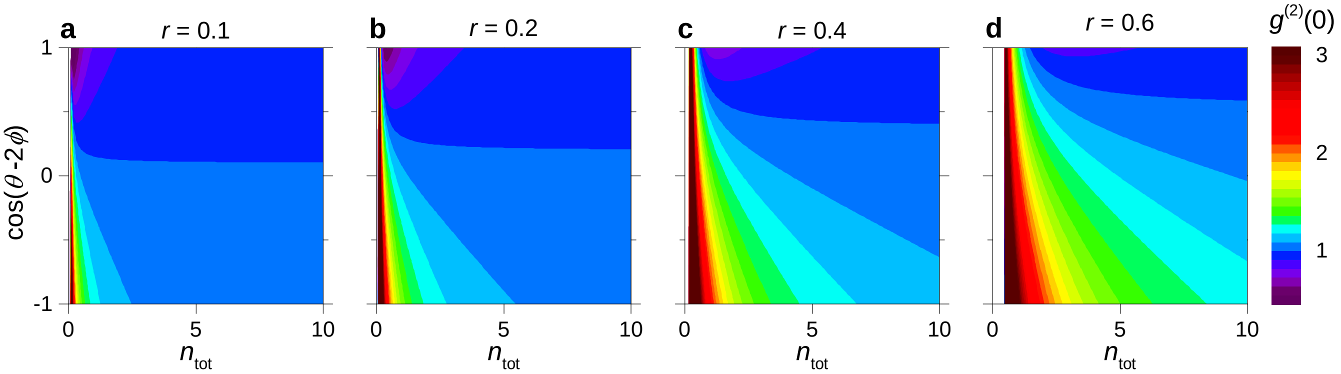

Supplementary Figure 2. **Second-order correlation function of a squeezed coherent state.** Map of the $g^{(2)}(0)$ function versus total population n_{tot} and $\cos(\theta - 2\varphi)$ for different amplitudes of the squeezing parameter. **a** $r = 0.1$, **b** $r = 0.2$, **c** $r = 0.4$, and **d** $r = 0.6$.

Low occupancies.

The most peculiar phenomenon expected to arise due to the weak Kerr nonlinearity is the photon antibunching. Even though some degree of antibunching is present almost for any initial conditions, a strong antibunching is strictly limited to the number of polaritons in a mode of about or less than unity. Other crucial parameters for the sizable antibunching are proper phase interrelation, namely $\cos(\theta - 2\varphi) \approx 1$, and relatively low squeezing amplitude (violet

region in Supplementary Fig. 2a-c). In this regime, $g^{(2)}(0)(t)$ is expected to oscillate between values below 1 and above 1 as shown in Ref. [1]. Considering our experimental layout, we estimate a polariton population in the range of a hundred at $t = 30$ ps, when the signal-to-noise ratio becomes insufficient to make any confident claims about the statistics of the emission. Indeed, the weakest light we can detect, which is defined by the low quantum efficiency and dark noise of the streak-camera, corresponds to about 50 polaritons in the system. From Equation (1) of the main text, the minimal detectable $g^{(2)}(0)$ at this point cannot be less than 0.98, which is clearly too high for confident claims of the antibunching.

This point can be further clarified by demonstrating the range of accessible $g^{(2)}(0)$ at every moment of time, as shown in Supplementary Fig. 3. Such estimation was done by calculating the $g^{(2)}(0)$ for the mode populations and squeezing amplitudes achievable in the experiment at every moment of time, as well as arbitrary values of $\cos(\theta - 2\varphi)$. As one can see, strong antibunching gets achievable only at $t > 40$ ps, which is beyond the scope of our experiment. At the same point, mode occupancies of the order of 1 or less can lead to the observation of superbunching with $g^{(2)}(0) > 3$, which would require $\cos(\theta - 2\varphi) \approx -1$ and large squeezing magnitudes (Supplementary Fig. 2d).

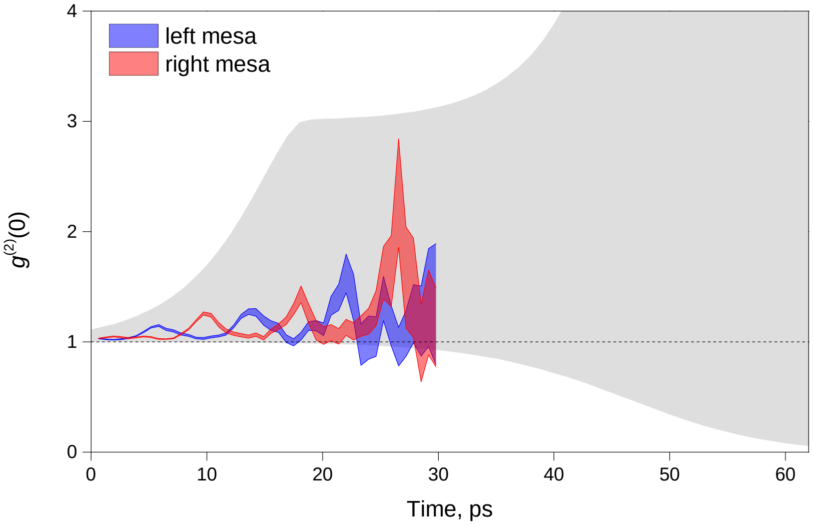

Supplementary Figure 3. **Achievable values of the second-order correlation function.** Gray area shows the values of $g^{(2)}(0)$ that can be achieved in the experiment for realistic squeezing amplitudes and arbitrary values of $\cos(\theta - 2\varphi)$. Experimental data from Fig. 2c of the main text are shown in red and blue.

High occupancies.

In the limit of the large occupancies, the second-order correlation is strictly limited to $1 - 1/\bar{\alpha}^2 < g^{(2)}(0) \leqslant 3$. For example, $r \to +\infty$ sets $g^{(2)}(0) \to 3$, which has been measured in Ref. [2]. This very mechanism limits the $g^{(2)}(0)$ with a value of 3 at $t < 30$ ps in our experiment, when the population of mesas is still large (Supplementary Fig.3).

The regime of the large field $n_{\text{tot}} \gg 1$ is exactly what we explore in our experiment, and as it can be seen from Supplementary Fig. 2, in this case the squeezing manifests itself in super-Poissonian light statistics. Here, the main signature of the squeezing is an increase of $g^{(2)}(0)$ when decreasing $\cos(\theta - 2\varphi)$. This holds for any value of squeezing and exactly corresponds to our observations presented in Fig. 2c-d of the main text. At the same point, the mechanism of generation of the super-Poissonian statistics differs significantly from the other observations of bunching in

polaritonic systems: it is the coupling of two nonlinear modes that induces the squeezing and the emission of bunched light.

In the regime of high occupancies, the increase of the maximal degree of bunching (height of peaks in Figure 2c of the main text) originates from the decrease of the mean occupation number n_{tot}, which defines the denominator of Equation (1) of the main text. This very mechanism is responsible for the gradual increase of the maximal reachable $g^{(2)}(0)$ during the first 20 ps in Supplementary Fig. 3. This trend stops at $g^{(2)}(0) = 3$, the maximal value allowed for the large occupancies. One should also note that such tendency fails at $n_{\mathrm{tot}} \sim 1$, where the decrease of n_{tot} can lead even to the decrease of the $g^{(2)}(0)$. Particularly, such an effect could be observed if one would reduce the population of the system while keeping constant $\cos(\theta - 2\varphi) \approx 1$ (Supplementary Fig. 2a), thus passing to the antibunching regime. More detailed discussion on this topic can be found in Ref. [1].

SUPPLEMENTARY REFERENCES

[1] Flayac, H. & Savona, V. Nonclassical statistics from a polaritonic Josephson junction. *Phys. Rev. A* **95**, 043838 (2017).

[2] Grosse, N. B., Symul, T., Stobińska, M., Ralph, T. C. & Lam, P. K. Measuring Photon Antibunching from Continuous Variable Sideband Squeezing. *Phys. Rev. Lett.* **98**, 153603 (2007).
